# Dimensions management of traffic big data for short-term traffic prediction on suburban roadways

**DOI:** 10.1038/s41598-024-51988-7

**Published:** 2024-01-17

**Authors:** Arash Rasaizadi, Fateme Hafizi, Seyedehsan Seyedabrishami

**Affiliations:** Faculty of Civil and Environmental Engineering, Tarbiat Modares University, Tehran, Iran

**Keywords:** Civil engineering, Scientific data

## Abstract

Since intelligent systems were developed to collect traffic data, this data can be collected at high volume, velocity, and variety, resulting in big traffic data. In previous studies, dealing with the large volume of big traffic data has always been discussed. In this study, big traffic data were used to predict traffic state on a section of suburban road from Karaj to Chalous located in the north of Iran. Due to the many and various extracted features, data dimensions management is necessary. This management was accomplished using principal component analysis to reduce the number of features, genetic algorithms to select features influencing traffic states, and cyclic features to change the nature of features. The data set obtained from each method is used as input to the models. The models used include long short-term memory, support vector machine, and random forest. The results show that using cyclic features can increase traffic state prediction's accuracy than the model, including all the initial features (base model). Long short-term memory model with 71 cyclic features offers the highest accuracy, equivalent to 88.09%. Additionally, this model's reduced number of features led to a shorter modelling execution time, from 456 s (base model) to 201 s.

## Introduction

The advent of intelligent transportation systems has revolutionized the data collection process. Traditional data collection methods, using human resources, collect data in a limited space and time and a small sample of the whole population. While using intelligent systems, these limitations are removed mainly^1^. One of these types of data is the collected traffic data of suburban roads of Iran, which are collected and reported the volume and average speed of traffic over time by using inductive sensors. Suburban traffic data is considered big data because it includes all three main big data properties: high volume, velocity, and variety^2^. Many observations and features of this data indicate a large volume. This data is collected over time in one-hour intervals, indicating its velocity. The diversity of data is also great in terms of the source of features. Big data analysis is more complex than other data but leads to more reliable results^3^.

After describing the big nature of suburban traffic data, it is necessary to find its applicability for the short-term prediction of traffic parameters. Short-term prediction of traffic parameters is one of the intelligent tools for traffic demand management. In this method, traffic parameters for the short-term future, generally considered from one hour to several months (less than a year), are predicted and provided to passengers and system operators^4^. Based on the predicted information, passengers can better plan their future trips, and the system operator can plan and implement short-term transportation network management policies^5^. The predicted traffic parameters include average speed^6^, traffic volume^7^, and traffic state^8^. The two average speed and traffic volume parameters are quantitative and contain integer numbers greater than zero. The traffic state parameter is a discrete parameter that generally includes light, semi-heavy, and heavy states. In choosing a predictive model, it is essential to match the model's output with the nature of the predicted parameter.

Yang et al.^9^ employ statistical models (i.e., space–time (ST) model including vector autoregressive (VAR), autoregressive integrated moving average (ARIMA), and machine learning models such as support vector machines (SVM), multi-layer perceptron (MLP), and recurrent neural network (RNN) to explore the influence of periodic component on short-term prediction of speed. Although both statistical models and machine learning models aim to predict traffic speed, they differ significantly in model structure and interpretation ability. In terms of the specific characteristics of these approaches, researchers need to select the corresponding model according to specific requirements and assumptions.

In the study by Cheng et al.^10^, to select the methods that most accurately predict travel mode choice, authors make a comprehensive comparison between random forest (RF), SVM, adaptive boosting (AdaBoost), and multinomial logit (MNL). Also, this comparison aims to see the differences between machine learning and statistical approaches. Among the four prediction methods that have been examined, it was concluded that RF and SVM have the most accurate prediction for their case study. However, RF is more computationally efficient than SVM and takes less execution time to train the model. In addition, unlike other machine learning approach working like a "black box", the relative importance of explanatory variables can be determined by the RF model. This is important for providing substantial insights into formulating appropriate and effective transport policies.

Wang and Ross^11^ explore applying the extreme gradient boosting (XGB) to model the travel mode choice and compare the result with an MNL. They conclude that the XGB has higher prediction accuracy than the MNL, especially when the dataset is not extremely unbalanced. The MNL model has great interpretability and explanatory power, and it also demonstrates strong consistency between training and testing error terms.

Bratsas et al.^12^ investigate on comparing the predicting power of machine learning models, including RF, SVM, and MLP using probe data of Thessaloniki, Greece. The case study results show that while the SVM model performs best at stable conditions with minor variations, the multi-layer perceptron model brings more accutrate prediction for greater variations.

Jiang et al.^13^ employe backpropagation neural network (BPNN), nonlinear autoregressive model with exogenous inputs neural network (NARXNN), support vector machine with radial basis function as kernel function (SVM-RBF), support vector machine with linear function (SVM-LIN), and MLR. Employed statistical models are also ARIMA, VAR, and ST. The statistical models have more theoretical interpretability, and machine learning models are more flexible with no or little prior assumptions of input variables. Also, machine learning models can process outliers, missing and noisy data. Through a case study in Beijing based, this study supplies practical applications of speed prediction and comparisons of prediction metrics.

Golshani et al.^14^ evaluate the performance of discrete, continuous, and joint discrete–continuous statistical models with the performance of the NN, as a ML model. The results indicate that besides superior prediction accuracy, the NN can capture nonlinearities, which suggests that it can also be more accurate to capture asymmetrical and nonlinear responses for policy analysis purposes.

The previous literature shows that machine learning algorithms have a better performance than statistical models to analyze big data and predict traffic parameters. However, in many cases, the large volume of observations and features in big data influence the performance of machine learning algorithms. In other words, one of the main challenges in the analysis of big traffic data is data management. In the big data preprocessing stage, data dimensions can be managed using techniques by reducing data dimensions, selecting features, and changing features. The importance of data processing and data management is so significant that many researchers have focused on this field. In some previous studies, dimensionality has maintained or increased accuracy.

Wu et al.^15^ use principal component analysis (PCA) to reduce the dimension of the pattern. This approach aims to solve the problems of slow matching speed and interference of irrelevant dimension. This problem caused by the high dimension of the pattern. Gao et al.^16^ introduce a hybrid approach based on PCA and wavelet neural network (WNN) for short-term prediction of traffic flow. The historical data of the predicated traffic volume has been processed by PCA first, and then the outputs of PCA form the input data of the WNN. Zhang and He^17^ also analyse a hybrid approach based on PCA and combined neural network (CNN) for short-term prediction of traffic flow. This approach reduces the dimension of input features. Gulacar et al.^18^ apply the PCA on the all of feature to reduce the dimensions of them and remove the noise. To remove the linear correlations between features, Zheng et al.^19^ also employ PCA.

Chen et al.^20^ present a sparse hybrid genetic algorithm (GA) that can choose whether a variables should be used to train the least squares support vector regression (LSSVR) for short-term traffic flow prediction. The results show that the proposed algorithm can brings accurate predictions with fewer variables. In some studies^21–23^, GA is employed in combination with neural networks to identify suitable hyper-parameters or optimize the initial parameters and weights of the neural networks.

Yao and Qian^24^ consider time features including week-of-year, month-of-year, day-of-week, and holiday. For the cyclic month and week of year features, they employe sine and cosine functions to transform them into a two-dimension vectors. An advantage of this "clockwise" encoding is that each variable is mapped onto a circle. The lowest value for that variable appears next to the largest value (e.g., January is next to December).

The previous literature shows the performance of data analysis techniques to predict traffic state. Current study is distinguished by compiling a traffic big data and its comprehensive management of big traffic data using principal component analysis (PCA), genetic algorithms, and cyclic features, as well as the implementation of diverse prediction models (LSTM, SVM, and RF).

In this study, the purpose of big data processing of the Karaj-Chalous road in the north of Iran is to predict the traffic state parameter. For this purpose, three preprocessing methods are used: principal component analysis to reduce the features, genetic algorithm to select the practical features, and cyclic features to change the nature feature. In the second step, three machine learning algorithms, including long short-term memory, random forest, and support vector machine, are trained using preprocessed and big unprocessed data. Accuracy metrics of traffic state prediction are evaluated separately by the model and data used. As a research hypothesis, it is expected that using data dimension management methods, the speed of modelling execution is reduced, and some of these methods maintain or increase the accuracy of traffic state prediction. This study also seeks to answer these two questions. What effect will the use of principal component analysis methods, cycle features, and genetic algorithms have on the accuracy of predictions? Which algorithm is more accurate than other machine learning algorithms, long short-term memory, random forest, or support vector machine?

The principal component analysis is chosen because it can reduce the complexity and noise of the data. Also, it can highlight the most important predictor variables and relationships. The Genetic algorithms which relying on biologically inspired operators are commonly used to generate high-quality solutions to optimization and dimension reduction problems. Using cyclical features is important in time series prediction because these features capture recurring patterns or oscillations within a data set.

This research tries to cover two research innovations. The first innovation is compiling a big database containing a variety of factors that influence the traffic state parameter. Beside of the variety, this big dataset has large volume and velocity. The second innovation of the research is using this big data and data management methods to predict the short-term parameter of traffic state, which has not been studied in previous studies.

## Data

**Figure 1:**
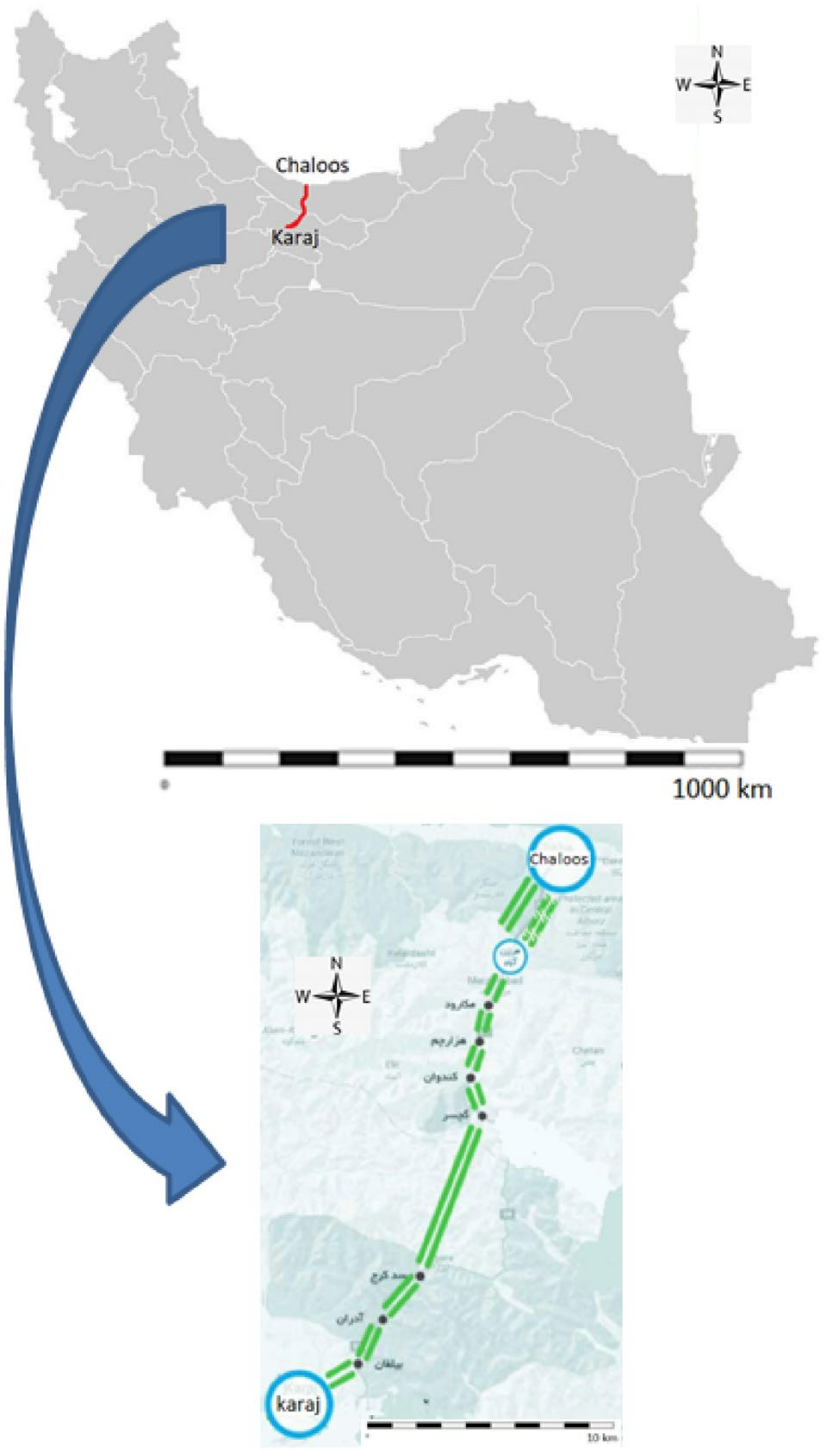
Map of Karaj-Chalous road located in the north of Iran (ArcGIS 10.8.2 and Microsoft PowerPoint 2020 are used to create the map).

The Karaj-Chalous road in the north of Iran is part of the route from Tehran as the capital of Iran to the country's northern coast. It is 170 km long. Along the Karaj-Chalous road, three parallel roads, with different lengths, connect Tehran with the cities of the north. This road experiences heavy traffic congestion during some days of the year. In general, finding the pattern of non-mandatory trips is more complicated than mandatory trips. This study focuses on the route from Karaj to Chalous in the Purkan section. The data were obtained from the Road Maintenance and Transportation Organization's website, which is freely accessible. A map of the Karaj-Chalous road can be seen in Fig. 1.

In this research, the hourly traffic state parameter is determined based on the ratio of average hourly speed to the free-flow speed and the ratio of hourly traffic volume to the hourly capacity of the Purkan section. Table 1 shows how to define this parameter, which includes three states: light (A), semi-heavy (B), and heavy (C). The Roads Maintenance and Transportation Organization have developed this method of defining the traffic state parameter.

**Table 1:** Definition of traffic state parameter.

The ratio of the speed to free-flow speed	The ratio of the volume to capacity					
	Less than 0.1	0.1–0.3	0.3–0.5	0.5–0.7	0.7–0.9	More than 0.9
More than 0.95	A	A	A	B	B	C
0.8–0.95	A	A	B	B	B	C
0.6–0.8	A	B	B	B	C	C
0.45–0.6	B	B	B	C	C	C
Less than 0.45	C	C	C	C	C	C

After determining the traffic state, defining the added features as predictors is necessary. These features were not present in the original raw dataset and were extracted and added to the data set during this research. Table 2 introduces these features.

**Table 2:** Definitions of the predictor features categories.

Feature category	Description	Content
Season	Including spring, summer, fall, and winter	4 dummy variables each can take values either 1 or 0
Solar month	Including 12 Solar months	12 dummy variables each can take values either 1 or 0
Lunar month	Including 12 Lunar months	12 dummy variables each can take values either 1 or 0
Day of a Solar month	Including 29–31 days of a Solar month	31 dummy variables each can take values either 1 or 0
Day of a Lunar month	Including 28–30 days of a Lunar month	30 dummy variables each can take values either 1 or 0
Time of day	Including 24 h a day	24 dummy variables each can take values either 1 or 0
day and night	Including two modes of day or night	can take values either 1 or 0
Holidays	Indicating an official holiday in the country	can take values either 1 or 0
Holiday type	Indicating the type of official holiday in the country	18 dummy variables each can take values either 1 or 0
Number of holidays	Number of consecutive holidays	Integers greater than or equal to zero
Days before holidays	Indicating a holiday in 3 next days (each is a feature)	Can take values either 1 or 0
Type of ahead holidays	Indicating the type of holiday in 3 next days (each is a feature)	18 dummy variables each can take values either 1 or 0
Days after holidays	Indicating a holiday in 3 past days (each is a feature)	can take values either 1 or 0
Type of previous holiday	Indicating the type of holiday in 3 past days (each is a feature)	18 dummy variables each can take values either 1 or 0
Blockage	Blockage due to traffic restrictions or natural disasters	Can take values either 1 or 0
Blockage of the opposite direction	Blockage of the opposite direction due to traffic restrictions or natural disasters	Can take values either 1 or 0
Blockage of the parallel route	Blockage of the parallel route due to traffic restrictions or natural disasters	Can take values either 1 or 0
weather	Including three modes of sunny, rainy, and snowy	3 dummy variables each can take 0 and 1

Since the traffic characteristics on the Karaj-Chalous road are significantly a function of holidays, a major part of Table 2 is related to the country's holidays. Also, due to the different nature of the holidays, 18 different types of holidays have been considered. For example, Nowruz, 14th and 15th Khordad, 21st Ramadan, Tasua, and Ashura are considered different types of holidays. The relationship between holidays and two lunar and Solar calendars has also led to considering both calendars.

**Figure 2:**
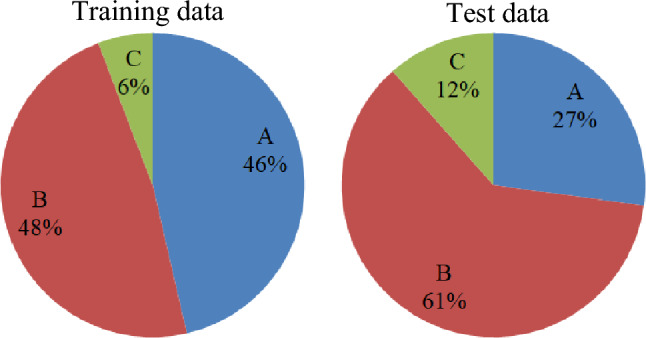
Relative frequency (percentage) of traffic states by training and test data.

A set of predictive features and the parameter of traffic situation has been collected during the five years from Farvardin 1, 1393 to 1398 (March 21, 2014 to 2019); the first four years have been used for training and the last year for testing models. So, it is expected that the dataset has large volume because of its large number of records and features. Figure 2 shows the frequency of traffic states by two training and test data.

Records of this dataset is generated every 1 h. It is a non-stop data collection means that the dataset has the velocity. Having large volume, variety and velocity is three essential factors for every big data. So, used traffic dataset has characteristics associated with big data.

## Methodology

Three parts make up the methodology of this study. The first part covers data dimension management methods. The second part reviews machine learning models. Lastly, evaluation indexes of traffic state prediction are discussed.

### Data dimension management

The data dimension management methods used include principal component analysis, cyclic features, and genetic algorithms discussed below.

#### Principal component analysis

Principal components analysis (PCA) is a well-known unsupervised dimensionality reduction technique. This technique constructs relevant variables through linear combinations of the initial variable. it does not require any prior assumptions and can also be easily implemented and interpreted. The PCA can identify the essential primary features in explaining the total variance. The PCA also determines the features with the largest share of the total variance. Output features of PCA can be calculated as linear combinations of primary features. The PCs are obtained based on the unit vector decomposition method in this research. The unit vector of each PC is called the eigenvector. An eigenvalue of a PC is the sum of the squares of observations distances from the origin. The high eigenvalue for each PC indicates the high variance of the observations relative to that PC and its importance in terms of the high share of explanations of the total variance^25^.

1 $$eigenvalue\, for\, PC1=\sum_{i=1}^{m}{d}_{i}^{2}$$

where *m* is the number of observations and ${d}_{i}$ is the observations distance of PC1 from the origin.

By using the PCA technique it is expected that the computational load can be reduced without a noticeable decrease in accuracy.

#### Genetic algorithm

GA can help determine the features that influence the traffic state and eliminate the unrelated features. The process of the used GA is as follows^26^:

Step 1: The population size for each generation (p), the probability of mutation (pm), and the stopping criterion are determined.

Step 2: The initial population of chromosomes is randomly selected.

Step 3: Repeat the following steps for each chromosome until the stopping criterion is met.

- A decision tree model is learned, and the fit of each chromosome is calculated.
- Two chromosomes are selected for each reproduction from 1 to p/2 based on the model's fit.
- Exchange genes are selected to produce two chromosomes from the combined genes.
- The chromosome is mutated with a probability of pm.
- End

In the above process, the chromosome, which contains the genes, is a vector of zero and one, which one indicates the existence and zero indicates the absence of a feature. The population consists of a set of chromosomes.

The prediction accuracy and running time of the GA technique can show the efficiency of it.

#### Cyclic features

The numbers are assigned to the season, month in the Solar calendar, month in the lunar calendar, day in the Solar calendar, day in the lunar calendar, day of the week, and hour change at certain intervals. On the other hand, these numbers are not absolute and cannot be compared mathematically. In such cases, a practical solution is to define the cyclic features using the change of the trigonometric variable. In this method, the values of each feature change on the perimeter of a circle, and the distances between the values are observed. Using Eqs. 2 and 3, we can change the initial features and generate the cyclic features^27^.

2 $${x}_{sin}={\text{sin}}\left(\frac{2*\pi *x}{{\text{max}}\left(x\right)}\right)$$

3 $${x}_{cos}={\text{cos}}\left(\frac{2*\pi *x}{\mathit{max}\left(x\right)}\right)$$

By using this method, the increase of accuracy can be expected or it least the it can preserve the accuracy by using less predictor features.

### Machine learning models

The used machine learning models include long short-term memory, support vector machine, and random forest, discussed below. These models were chosen for their strengths in handling complex relationships, temporal dependencies, and varied features, providing a comprehensive analysis of the research objectives.

#### Long short-term memory

The input of the LSTM model includes short-term memory, long-term memory, and some observations in the training dataset. This model has four gates. The long-term input of the model enters the forget gate, and this gate decides which of the irrelevant inputs to remove (Eq. 4). The short-term input of the model and some observations of the training data enter the training gate, and this gate decides which input to learn (Eq. 5). The information passed, including the outputs of the forget gate and the training gate, enters the reminder gate. The output of this gate is new long-term memories (Eq. 6). Finally, the output gate also updates the short-term memories and produces the final output of the model (Eq. 7)^28^.

4 $${f}_{t}=\upsigma \left({w}_{f}\left[{h}_{t-1},{x}_{t}\right]+{b}_{f}\right)$$

5 $${l}_{t}={\text{tanh}}({w}_{n}\left[{h}_{t-1},{x}_{t}\right]+{b}_{n})$$

6 $${r}_{t}={l}_{t}+{f}_{t}$$

7 $${o}_{t}=\upsigma ({w}_{o}\left[{h}_{t-1},{x}_{t}\right]+{b}_{o})$$

In the above equations,${f}_{t}$, ${l}_{t}$, ${r}_{t}$ and ${o}_{t}$ are the factors of the forget gate, training gate, reminder gate, and output, respectively. σ is the sigmoid function, and ${w}_{x}$is the weight vector of gate neurons x. ${h}_{t-1}$ is the output of the previous block, ${x}_{t}$ is the input of gate x at time t, and ${b}_{x}$ is the deviation of gate x.

The model's hyperparameters and their values in this study are as follow:

Number of LSTM layers: 20

Number of neurons in each LSTM layer: equals to total number of traffic states (c)

Activation function: Hyperbolic tangent (tanh)

Dropout: 0.2

Batch size: 32

Epochs: 500

It is supposed that the LSTM is compatible with the big data analysis and brings high accuracy predictions.

#### Support vector machine

The SVM aims to find the best boundary between the classes to have the most significant possible distance from all classes. To determine the best boundary and construct an optimal classification, it calculates the distance of the boundaries obtained with the support vectors of each category. Finally, it selects the boundary furthest from the existing classes. If the data are nonlinear, the data needs to be mapped to another space using the kernel mathematical function in which the data is separable. In this study, the radial basis function (RBF), according to Eq. 8, has been used as the kernel function^29^.

8 $$K\left({X}_{i},X\right)=exp(\frac{{\Vert {X}_{i}-{X}_{j}\Vert }^{2}}{2{\sigma }^{2}})$$

where σ is the free parameter of the model and ${\Vert {X}_{i}-{X}_{j}\Vert }^{2}$ is the square of the Euclidean distance between the two feature vectors ${X}_{i}$ and ${X}_{j}$.

The model's hyperparameters and their values in this study are as follow:

Kernel: Radial basis function kernel

C (Regularization parameter): 0.01

Gamma: 10

The SVM model performance we be analyzed based on its accuracy and running time.

#### Random forest

The RF model consists of a large number of decision trees. In this model, the training data is divided among the decision tree models separately, and after training them, a prediction is made for each decision tree model. The most repetitive prediction is determined as the overall prediction of the RF model. The following steps show how the RF algorithm works^30^.

Step 1: Random samples of the training data set are taken based on the number of decision tree models considered.

Step 2: Using each sample, a decision tree model is trained.

Step 3: Prediction of each decision tree model is made for the test data.

Step 4: Using averaging or voting methods, the repeated label is selected and reported as the final prediction.

The decision tree model starts with one node, and the initial node is connected to the next nodes using branches. This study uses the entropy relationship (Eq. 9) to determine how the branches develop.

9 $$Entropy=\sum_{i=1}^{c}-{p}_{i}*{{\text{log}}}_{2}(pi)$$

where ${p}_{i}$ is the relative frequency of the traffic state i and c is the total number of traffic states.

The model's hyperparameters and their values in this study are as follow:

Number of randomly drawn candidate variables (mtry):$\sqrt{c}$

Node size: 1

Number of trees: 1000

Splitting rule: Gini impurity

It supposed that the RF achieves more accurate predictions compared to single decision forests.

### Models evaluation

After data training, the prediction accuracy of the models and F_1_ is calculated using test data through Eqs. 10 to 13^31^.

10 $$Prediction\,accuracy=\frac{The\,number\,of\,observations\,that\,have\,been\,correctly\,predicted}{Total\,number\,of\,observations}$$

11 $$Precision=\frac{True\,Positive}{True\,Positive+False\,Positive}$$

12 $$Recall=\frac{True\,Positive}{True\,Positive+False\,Negative}$$

**Figure 3:**
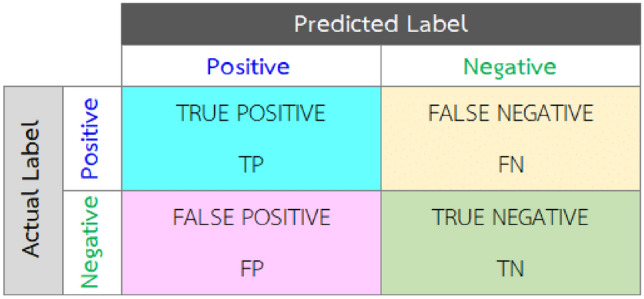
Confusion matrix^8^.

True positive and false negative definition is presented in Fig. 3.

13 $${F}_{1}=\frac{2(Precision*Recall)}{Precision+Recall}$$

Also, another criterion used to compare the models is modelling execution time in seconds. Data dimension management algorithms are expected to reduce modelling execution time.

## Results

**Figure 4:**
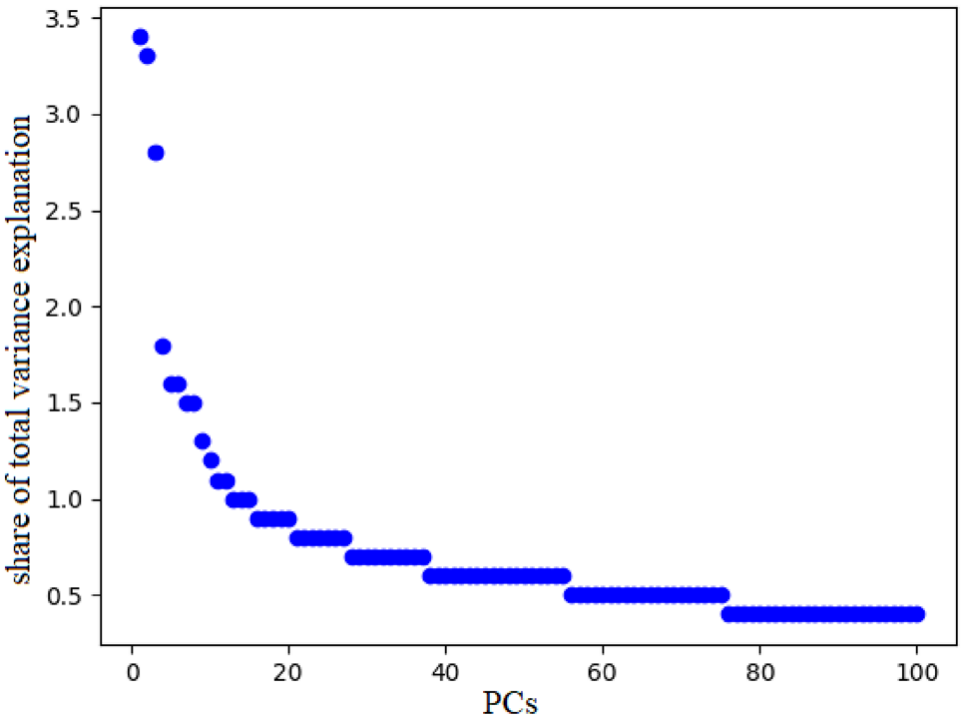
The share of each PC in the total variance of the initial properties.

The data dimension management methods are used to reduce the features as input of the models. The initial features listed in Table 2 are primarily nominal or ordinal. Therefore, they must be defined as dummies to be used in modelling. After defining the dummy features, the total number of features in the data set reaches 178. Figure 4 is used to select the number of PCs. The horizontal axis of this chart is the number of PCs, and the vertical axis is the variance share of each PC from the total variance. After the 30th component, the intensity of the changes decreases, so the 30 PCs are selected with 37% of the explanatory of the total variance.

After implementing the GA, the following features were not selected to predict the traffic state:

- Type of holiday in next day
- Holiday in the past day
- Holiday in the next day
- Holiday in third next day
- Blockage
- Blockage of the opposite direction
- Blockage of the parallel route

**Figure 5:**
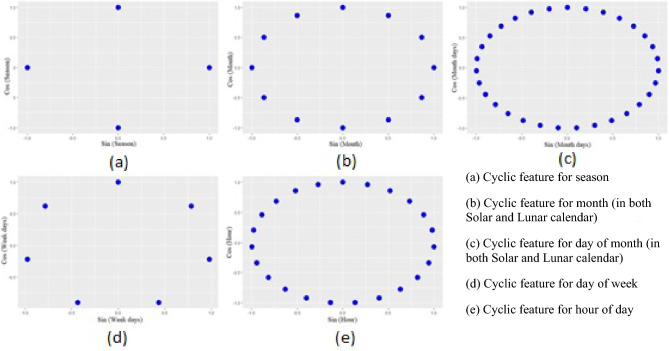
Cyclic features.

The definition of cyclic features is also shown in Fig. 5.

Table 3 shows the number of features extracted from each data dimension management method.

**Table 3:** Number of features extracted from each data dimension management method.

Method	Initial data set	Principal component	Selected features of genetic algorithm	Cyclic features
Number of features	187	30	137	71

Table 3 shows the PCA method with the least number of features and the GA with the highest number of features.

After training machine learning models using a set of different features, including all features (base), PCs, cyclic features, and selected features of the GA, the prediction accuracy and F_1_ of models are calculated and reported in Table 4 and 7, respectively.

**Table 4:** Prediction accuracy by model and feature set used.

Model	All features	PCs	Selected features of GA	Cyclic features
SVM	76.78	71.48	74.73	79.24
LSTM	85.81	78.82	84.27	88.09
RF	79.21	74.75	78.29	83.51

Table 4 shows that PCs reduce the accuracy of all three models than the base models trained with all the initial features. The use of selected features of the GA has approximately maintained the accuracy of the models, and it has been reduced only by about 1 to 2%. It is important to note that cyclic features increase the accuracy of the models. The accuracy of all three models has been increased by about 3 to 4% by using cyclic features. The difference in the effectiveness of data management methods goes back to their functional nature. The 30 extracted components explain only 37% of the total variance in the PC method. In the genetic algorithm, some features have also been removed. The use of cyclic features does not eliminate the features, but by changing them, the nature of the time-related features is defined as cyclical, while there is no need to define dummy features.

Table 4 also shows that based on all four categories of features used, the prediction accuracy of the LSTM model is higher than the other two models. The SVM model also results in the lowest accuracy in traffic state prediction. This is because SVM algorithm is not suitable for large data and does not perform very well with noisy dataset. The deep architecture of the LSTM model and the consideration of short-term and long-term correlation of observations on the one hand and the ability of this model to analyze big data, on the other hand, have led to greater accuracy than other models. Finally, the highest accuracy of traffic state prediction was obtained using the LSTM model and the cyclic features set, equivalent to 88.09%.

Based on Tables 5, 6, 7, and 8 all of the models have more accurate predictions for state B because of its high frequency in train and test datasets. Predicting traffic state C has the least accuracy because it has the least frequency in train and test datasets. Using cyclic features increases the F1 for all of the states and for all of the models. On average, by using cyclic features compared to using all features, the F1 of predicting state A is increased by 6.5%, the F1 of predicting state A is increased by 4.4% and the F1 of predicting state A is increased by 7.9%. It is important that these features can enhance the F1 of state C, as a less observed traffic state, more than the other two states. The F_1_ is selected to evaluate models because it can handle imbalanced data sets. This metric provides a single and consistent measure of performance of different models.

**Table 5:** Recall by model and feature set used.

Model	State	All features	PCs	Selected features of GA	Cyclic features
SVM	A	0.77	0.65	0.71	0.81
	B	0.86	0.8	0.89	0.88
	C	0.56	0.49	0.55	0.63
LSTM	A	0.87	0.78	0.81	0.89
	B	0.92	0.81	0.85	0.96
	C	0.71	0.62	0.61	0.69
RF	A	0.82	0.72	0.82	0.83
	B	0.89	0.84	0.86	0.91
	C	0.68	0.53	0.59	0.67

**Table 6:** Precision by model and feature set used.

Model	State	All features	PCs	Selected features of GA	Cyclic features
SVM	A	0.69	0.58	0.62	0.78
	B	0.8	0.71	0.72	0.83
	C	0.49	0.4	0.45	0.51
LSTM	A	0.79	0.68	0.72	0.83
	B	0.87	0.69	0.74	0.89
	C	0.58	0.49	0.53	0.63
RF	A	0.69	0.64	0.71	0.77
	B	0.79	0.75	0.78	0.88
	C	0.52	0.45	0.57	0.66

**Table 7:** F_1_ by model and feature set used.

Model	State	All features	PCs	Selected features of GA	Cyclic features
SVM	A	0.73	0.61	0.66	0.79
	B	0.83	0.75	0.8	0.85
	C	0.52	0.44	0.5	0.56
LSTM	A	0.83	0.73	0.76	0.86
	B	0.89	0.75	0.79	0.92
	C	0.64	0.55	0.57	0.66
RF	A	0.75	0.68	0.76	0.8
	B	0.84	0.79	0.82	0.89
	C	0.59	0.49	0.58	0.66

**Table 8:** Modeling execution time (in seconds) by model and feature set used.

Model	All features	Principal component	Selected features of genetic algorithm	Cyclic features
SVM	918	135	630	326
LSTM	456	79	375	201
RF	402	56	270	148

Table 8 shows the modelling execution time of machine learning models by using features in seconds. R programming software and the eighth-generation core i7 processor with 16 GB of RAM have been used to execute the code.

Few PCs have led to the shortest in terms of the modelling execution time. The modelling execution time is directly related to the number of used features. Although using the GA and PCA decrease the accuracy of prediction (compared to using all features) but they have fewer modeling execution time. This benefit is more significant when we want to run online model and have processing power and time limitations. So, we can choose GA and PCA for dimension reduction regarding to the benefit of less execution time and cost of less prediction accuracy. The important finding is that using cyclic futures outperforms the base model (using all features) in terms of both modeling execution time prediction accuracy.

The execution time of RF and SVM models are the minimum and maximum execution times, respectively. In general, while having the highest accuracy in traffic state prediction, the LSTM model trained with cyclic features ranked fifth out of all twelve trained models regarding modelling execution time, which seems acceptable.

## Conclusion

Short-term prediction of traffic states on suburban roads and informing it by advanced traveller information systems can help transportation demand management. This is crucial for reducing peak-hour traffic congestion and optimizing the utilization of transportation networks. Such forecasts enable travelers to make informed judgments regarding their journey, including departure time and route selection. In addition, it has far-reaching implications, such as improving safety management, optimizing cargo transportation, efficiently scheduling public fleets, planning road maintenance, and implementing effective management policies. It has been done by analyzing big traffic data of suburban roads of Iran and using machine learning methods in this study. There are many features extracted and added to the data set, so the dimension of used big data expands and makes the modelling task more difficult. As a result, data dimension management is essential. According to the results, the three methods of managing data dimensions, such as PCA, cyclic features, and GA, reduce modelling execution time (relative to modelling execution time with all features). PCA method reduces prediction accuracy, while GA maintains it roughly. The use of cycle features is the only way to increase accuracy. Also, the LSTM model has a higher prediction accuracy than RF and SVM. Finally, the LSTM model with cyclic features has the highest accuracy of predicting the traffic state parameter for the suburban road of Karaj to Chalous, which is equivalent to 88.09%.

This study has a significant limitation due to the lack of access to certain traffic conditions variables, such as accidents on suburban roads, humidity and fog, and weather-related factors like ice and slippery roads, which are recorded by organizations like Traffic Police and Meteorological Organization, but their publication for research purposes is limited. For this reason, the lack of access to this information is considered as a limitation.

To continue this research, alternative data processing methods like backward feature elimination algorithms and convolutional neural networks can reduce the number of features.

## Supplementary Information

Supplementary Information.

## Data Availability

The dataset used and analyzed during this study are included in this published article.
